# Improving evidence-based primary care for chronic kidney disease: study protocol for a cluster randomized control trial for translating evidence into practice (TRANSLATE CKD)

**DOI:** 10.1186/1748-5908-8-88

**Published:** 2013-08-08

**Authors:** Chester H Fox, Bonnie M Vest, Linda S Kahn, L Miriam Dickinson, Hai Fang, Wilson Pace, Kim Kimminau, Joseph Vassalotti, Natalia Loskutova, Kevin Peterson

**Affiliations:** 1 Department of Family Medicine, State University of New York – University at Buffalo, 77 Goodell St, Buffalo, NY 14203, USA; 2 Department of Family Medicine, University of Colorado School of Medicine, Aurora, CO, USA; 3 Department of Health Systems, Management, and Policy, Colorado School of Public Health, University of Colorado Denver, Denver, CO, USA; 4 American Academy of Family Physicians National Research Network, Leawood, USA; 5 Department of Family Medicine, University of Kansas School of Medicine, Kansas, USA; 6 Division of Nephrology, Mount Sinai Medical Center, New York, USA; 7 National Kidney Foundation, New York, USA; 8 Department of Family Medicine and Community Health, University of Minnesota Medical School, Minneapolis, Minnesota, USA

**Keywords:** Chronic kidney disease, Practice based research networks, Practice facilitation, Academic detailing, Computer decision support

## Abstract

**Background:**

Chronic kidney disease (CKD) and end stage renal disease (ESRD) are steadily increasing in prevalence in the United States. While there is reasonable evidence that specific activities can be implemented by primary care physicians (PCPs) to delay CKD progression and reduce mortality, CKD is under-recognized and undertreated in primary care offices, and PCPs are generally not familiar with treatment guidelines. The current study addresses the question of whether the facilitated TRANSLATE model compared to computer decision support (CDS) alone will lead to improved evidence-based care for CKD in primary care offices.

**Methods/Design:**

This protocol consists of a cluster randomized controlled trial (CRCT) followed by a process and cost analysis. Only practices providing ambulatory primary care as their principal function, located in non-hospital settings, employing at least one primary care physician, with a minimum of 2,000 patients seen in the prior year, are eligible. The intervention will occur at the cluster level and consists of providing CKD-specific CDS versus CKD-specific CDS plus practice facilitation for all elements of the TRANSLATE model. Patient-level data will be collected from each participating practice to examine adherence to guideline-concordant care, progression of CKD and all-cause mortality. Patients are considered to meet stage three CKD criteria if at least two consecutive estimated glomerular filtration rate (eGFR) measurements at least three months apart fall below 60 ml/min. The process evaluation (cluster level) will determine through qualitative methods the fidelity of the facilitated TRANSLATE program and find the challenges and enablers of the implementation process. The cost-effectiveness analysis will compare the benefit of the intervention of CDS alone against the intervention of CDS plus TRANSLATE (practice facilitation) in relationship to overall cost per quality adjusted years of life.

**Discussion:**

This study has three major innovations. First, this study adapts the TRANSLATE method, proven effective in diabetes care, to CKD. Second, we are creating a generalizable CDS specific to the Kidney Disease Outcome Quality Initiative (KDOQI) guidelines for CKD. Additionally, this study will evaluate the effects of CDS versus CDS with facilitation and answer key questions regarding the cost-effectiveness of a facilitated model for improving CKD outcomes. The study is testing virtual facilitation and Academic detailing making the findings generalizable to any area of the country.

**Trial registration:**

Registered as NCT01767883 on clinicaltrials.gov NCT01767883

## Background

Chronic kidney disease (CKD) and end stage renal disease (ESRD) are steadily increasing in prevalence in the United States (US) [1]. In 2000, there were 26 million American adults with CKD, and this represented a 30% increase over the past decade [1]. The annual incidence of ESRD is projected to increase from its 2007 level of 111,000 to 143,000 by 2020, when the total US population of people with ESRD is expected be over 770,000 [2,3]. The aging of the population and the rising prevalence of obesity, hypertension, and type 2 diabetes—the major risk factors for CKD—contribute to this trend [4]. CKD is a serious condition; stage three CKD is associated with a five-year all-cause mortality rate of 24.3% and a five-year need for renal replacement of 1.3%. Stage four CKD is associated with a five-year 45.7% mortality rate and a five-year 19.9% requirement for renal replacement [5].

While there is reasonable evidence that specific activities can be implemented by primary care physicians (PCPs) to delay CKD progression and reduce mortality [6-12], CKD is under-recognized and undertreated in primary care offices [13-15] and PCPs are generally not familiar with treatment guidelines [13,14]. Even when diagnosed, CKD is a chronic condition that, like diabetes, is frequently associated with co-morbidities, making effective treatment difficult due to the complexity of care.

The reasons for the slow rate of progress in improving healthcare delivery, especially for chronic conditions such as CKD, are complex. Rogers [16], in his classic studies of diffusion of innovation, outlined several potential barriers to adoption of innovation in many industries. These included lack of time and monetary resources, as well as organizational inefficiencies [16]. The health sector has been notoriously slow to adopt new approaches to care [17]. Competing demands and time limitations often inhibit the ability of PCPs to adopt evidence-based preventive services and manage chronic diseases well [18,19], and it takes several years for known evidence to become common practice [20-22].

The Chronic Care Model has been widely accepted and utilized in primary care practices as a means of improving evidence-based care [23,24]. Computer decision support (CDS) tools generated through electronic medical records and middleware vendors have been one important element of this model as a solution to some of the problems of implementing evidence into practice. However, a review by Garg *et al.* found that while CDS improved physician behavior in 73% of the studies, clinical markers were only improved 42% of the time [25-31]. Peterson *et al.* developed a nine-point action plan, including CDS and practice facilitation for implementing the Chronic Care Model [32]. This plan is referred to as TRANSLATE. TRANSLATE stands for ‘set your **T**arget, use **R**egistry and **R**eminder systems, get **A**dministrative buy-in, **N**etwork Information systems, **S**ite coordination, **L**ocal Physician Champion, **A**udit and feedback, **T**eam approach, and **E**ducation’ [32]. The combined efforts of the TRANSLATE model were highly effective in improving diabetes care in a randomized control trial (RCT) involving 24 practices and 8,405 diabetic patients [32]. At 12 months, intervention practices had significantly greater improvement in achieving recommended clinical values for systolic blood pressure (SBP), Hemoglobin A1C (HbA1C), and LDL cholesterol than control practices. Control practices were provided with a report of their process and outcome measures at baseline and were encouraged to continue usual quality improvement but did not receive CDS at the point of care [32].

The current study addresses the question of whether an adaptation of the facilitated TRANSLATE model with CDS, compared to CDS alone, will lead to improved evidence-based care for CKD in primary care offices, thereby slowing the progression to ESRD and improving patient health outcomes. A cluster randomized design was chosen to minimize contamination across arms and due to the logistical impossibilities of separating out practice workflow by provider to provide CDS and/or practice facilitation for only some providers within the same medical practice.

### Specific aims

#### ***Specific aim 1***

Conduct a cluster randomized controlled trial of point-of-care CDS plus the full TRANSLATE model of practice change, versus CDS alone in promoting evidence-based care in primary care practices for all patients with an eGFR <60 and >15 ml/min/1.73 m^2^ confirmed with repeat testing over three or more months. (CKD stages three and four).

#### ***Hypothesis 1.1***

CDS practices using the TRANSLATE model will provide a greater degree of evidence-based guideline-concordant care for CKD than CDS-only practices.

### Specific aim 2

Conduct an intent-to-treat and process analysis between the CDS practices with facilitation versus the CDS-only practices of the clinical outcomes of CKD progression and all-cause mortality.

#### ***Hypothesis 2.1***

Patients with stage three and four CKD in facilitated practices will have slower CKD progression than patients in CDS-only practices.

#### ***Hypothesis 2.2***

Patients with stage three and four CKD in facilitated practices will have significantly lower all-cause mortality than stage three and four patients in CDS-only practices.

#### ***Hypothesis 2.3***

The process evaluation will determine through qualitative methods the fidelity of the facilitated TRANSLATE program; find the challenges and enablers of the implementation process, and the contextual factors that contribute to TRANSLATE decisions and strategies; and translate lessons learned into pragmatic ‘best practices’ for future facilitation and dissemination.

### Specific Aim 3

Conduct a cost-effectiveness analysis that will compare the benefit of the intervention of CDS alone against the intervention of CDS plus TRANSLATE (practice facilitation) in relationship to overall cost per quality adjusted years of life.

#### ***Hypothesis 3.1***

The intervention of CDS plus TRANSLATE is more cost-effective than the intervention of CDS alone.

## Methods/design

This protocol consists of a cluster randomized controlled trial (CRCT) followed by a process and cost analysis of the study. The intervention consists of providing CKD-specific CDS versus CKD-specific CDS plus practice facilitation [33] for all elements of the TRANSLATE model.

The study is being performed by the State University of New York – University at Buffalo (UB) in collaboration with the American Academy of Family Physicians National Research Network (AAFP/NRN) with primary care practices across the United States. Ethical approval was obtained by the Institutional Review Boards at both UB and AAFP/NRN for all aspects of the study.

### Recruitment and randomization procedures

#### ***Inclusion criteria for clusters***

Only practices providing ambulatory primary care as their principal function, located in non-hospital settings, employing at least one primary care physician, with a minimum of 2,000 patients seen in the prior year, are eligible. ‘Practices’ are defined as distinct office locations that belong to organizations with one or more practice sites. Candidate practices are drawn from members of the Distributed Area Research and Therapeutics (DARTNet) members. These members have point of care decision support for preventive care and multiple chronic diseases. CKD will be added to the chronic disease management reminder system.

#### ***Inclusion criteria for patients***

Patient-level data will be drawn from the population of all active patients at each practice and will include all patients diagnosed with stage three CKD. Patients are considered to meet stage three CKD criteria if at least two consecutive eGFR measurements at least three months apart fall below 60 ml/min. At the inception of the study, we used data from the preceding 12 months to identify eligible patients (eGFR <60 and >15 ml/min) with additional patients added to the analytical dataset as they meet study criteria. These criteria were also used to initiate the clinical decision support algorithms.

#### ***Recruitment***

Recruitment for the study began in April 2012. The recruitment goal is to enroll 36 practices total, randomized into the two study arms. To date, 29 practices have been enrolled in the study. 25 of these practices have been randomized in two waves. The intervention period for the first wave of 18 practices began in January of 2013. The intervention for the second wave of seven practices will begin in June of 2013. We plan to recruit and randomize all additional practices by the end of 2013. Consent was obtained at multiple levels; a practice administrator signed a practice agreement and data use agreement for each individual practice organization. From each individual practice site (cluster), informed consent was obtained from a site coordinator and a lead physician at each site, as representatives of the cluster. Consent was obtained prior to randomization.

#### ***Randomization protocol***

In cases where two or more selected practices were drawn from the same multi-practice organization, contamination was limited by checking for clinician overlap. If overlap was minimal, organization was used as a stratification variable; if overlap was potentially problematic in terms of contamination, practices were constrained to be in the same group. From the pool of available practices, pre-study information from electronic health record (EHR) data on baseline performance characteristics related to CKD or in general and variables which may influence outcome measures, including practice and patient panel characteristics as well as pre-study clinical measures were collected (full-time equivalent physicians, mean HbA1c, % diabetic, % stage four CKD, % of diabetics with HbA1c >9, mean GFR, mean SBP, % with SBP >130, % with SBP >140, % African American, % Hispanic, % uninsured) [34]. Stratification variables included geographic region and organization. All possible combinations of the 18 eligible practices into two groups of nine were generated [35] using the IML procedure in SAS [36], retaining order so that the first designated group would be assigned to the intervention, the remaining practices to controls (n = 48,620). Only randomizations that were balanced on stratification variables were retained (n = 1,728). For each randomization, a balance criterion (defined as the sum of squared differences on standardized variables between control and intervention groups) was computed. After examining the distribution of the balance criterion, a maximum allowable difference between the groups was established and an optimal set of randomizations identified (n = 136). From this set, one was chosen using a random number generator and practices were assigned to treatment or control arms. This process was repeated for the next group of seven practices, including a dummy practice in the randomization procedure to accommodate the uneven number of practices. The remaining practices will be randomized using the same protocol. As with all CRCTs, patients are not randomized *per se*; they are assigned to treatment conditions along with the medical practices in which they receive care.

#### ***Power analysis***

Given the CKD national prevalence rate of 13% and an expected average of 5,500 active patients per practice, we expect roughly 715 CKD patients per practice (this estimate is supported by a recent analysis of >110,000 individuals with stage three or four CKD drawn from over 100 DARTNet practices.) With 18 practices per arm and a minimum of 200 patients per practice (smallest practice estimated at 2,000 patients with a conservative 10% prevalence of CKD over life of study) there will be a minimum of 3,600 patients per arm (actual expected sample size is over 14,000 per arm.) A sample size of 3,600 per arm will provide >80% power to detect a 0.18 effect size difference between two arms at a single time point if the intra-class correlation (ICC) is 3%. This effect size was assumed based on previous results from the diabetes TRANSLATE study [32]. In terms of change over time, a sample size of 3,600 will provide >80% power to detect a small linear trend effect, increasing from 0 at baseline to 0.2 standard deviation (SD) at final follow up, with four observations per person and an ICC of 3%, with a random effects structure with random intercept and random slope [37].

#### ***Intervention design***

Four elements of the TRANSLATE method will be implemented in both groups, while the remaining ones will apply to facilitated practices only. All interventions will occur at the cluster level, and there will be no randomization or specific intervention undertaken for individual patients. The CDS-only practices will have CKD decision support algorithms based on the Kidney Disease Outcomes Quality Initiative (KDOQI guidelines added to their existing CDS. There will be three separate vendors that will be providing point of care support for the study. Computer Integrated Networks of America (CINA), Health Metrics Systems (HMS), and an EMR with integrated CDS Medent. All practices will receive one session of introductory academic detailing [38] concerning the rationale for the algorithms. They will also be provided related technical support on request. Facilitated practices will receive the CKD-specific CDS, as well as a practice facilitator to assist them in the implementation of the full TRANSLATE model (Table 1).

**Table 1:** Translate elements by intervention arm

**TRANSLATE elements that will be used in both arms**	
**T**arget:	Common targets will be set for all practices and tracked through the CKD tool. The CDS-only practices will receive a quick reference guide for the treatment of CKD
**R**egistry and reminder systems	CINA created a CKD registry and will maintain it throughout the study period. Point-of-care decision support specific to CKD will be provided to practice staff and physicians prior to patient visits.
**A**dministrative buy-in	Obtain consent from each practice and all practice sites asked to identify a physician champion and site coordinator to oversee study implementation at their site.
**N**etwork Information systems	The information systems (EHRs and CDS) will be used to create system level reports across all practices.
**TRANSLATE element that will be used only in facilitated CDS practices**	
**S**ite coordination	A site coordinator at each practice will assemble a quality improvement (QI) team that will meet monthly to review performance data regarding CKD. The site coordinator will also work with the clinicians and practice staff to implement workflow changes such as pre-visit planning, standing orders, and patient education materials to improve efficiency of disease management. In addition, the site coordinator will be in contact with the practice facilitator by videoconference for assistance and advice.
**L**ocal physician champion	This person will be the clinician leader and educator for other providers in each practice. Responsibilities will include supporting the site coordinator and the QI team. This physician will be in contact with the academic mentor for the practice regarding clinical questions about CKD and will participate in learning collaboratives with the site coordinator.
**A**udit and feedback	Practice, individual provider, and patient-level outcome reports for the intervention practices will be generated through CINA regarding the seven performance measures (BP, HbAIC, LDL, use of ACE/ARB, referral to a nephrologist, smoking cessation and avoidance of NSAID or Cox-2) and will be reviewed by the team. Reports will also be reviewed quarterly with the practice facilitator by videoconference. The videoconference will allow the facilitator to learn what worked in each practice and to share what other practices have implemented successfully.
**T**eam approach	A quality improvement (QI) team consisting of the local physician champion, site coordinator and nursing, front office, and administrative staff will meet monthly to review progress of the CKD project. Workflow changes will be recommended and tested.
**E**ducation	An educational program using academic detailing and practice facilitation and videoconferencing will be utilized to support the practices’ efforts. All facilitated practices will be assigned an academic practice mentor. This mentor will be available to the office physician champion and practice coordinator to answer any questions and discuss plans. The academic mentor will review the practice’s data and participate in a quarterly videoconference with either the study coordinator or the lead clinician to review progress on the project.

#### ***Facilitation protocol***

The facilitators' overall role is to help the practices implement the TRANSLATE model to improve guideline-concordant care for CDK. The facilitator will assist with the site coordination, physician champion's needs, audit and feedback, team approach, and education. Two practice facilitators are each assigned to half of the CDS+ facilitation practices. Throughout the course of the intervention period, the facilitators will engage in virtual interactions with the practices, in which they will work with practice quality improvement (QI) teams to identify and solve issues specifically related to their care of patients with CKD and form and maintain relationships with each practice. Facilitation objectives include: assisting practices in setting goals to implement CKD guidelines; helping practice teams, strategize, test, and implement change; facilitating meetings and fostering a continuous QI culture; serving as a liaison for data and performance feedback; and sharing best practices and linking intervention practices.

Due to the national scope of this project, facilitation contacts will occur ‘virtually’ using videoconferencing (GoToMeeting®), phone, and e-mail. General communication will entail a minimum of once monthly teleconferences with each practice’s site coordinator to review the status of the project, monthly virtual QI team meetings, monthly calls between the academic mentors and the lead physician, periodic learning collaboratives, and a benchmarking webinar every six months with the academic detailing team.

### Data collection

#### ***Clinical data***

From practice EHRs, we will collect the measurements outlined in Table 2. Medication fulfillment data will be collected from Surescripts RxHub through CINA or the practice EHR. Death will be determined from information in practice EHRs or from linkages to the National Death Registry. We will check for deaths among patients who have not made any visits in the prior 12 months in the first two years of the intervention and in the prior six months in the final year of the intervention. Medicare claims data will be obtained before and after the intervention to allow for a proper cost analysis.

**Table 2:** Clinical data elements

**Data element**	**Measure type**
Year of birth	Numerical
Gender	M/F
Race/ethnicity	Standard major groups and Other
Current smoking	Current, never, past
Height and weight/BMI	Hgt, wgt actual
Total visits/encounters	Encounter records
Hemoglobin	Numerical result
HDL	Numerical result
LDL-C	Numerical result
Triglycerides	Numerical result
Creatinine	Numerical result
AST	Numerical result
ALT	Numerical result
HbA1c	Numerical result
25 OH Vitamin D	Numerical result
Electrolytes	Numerical result
Serum phosphorous	Numerical result
PTH intact	Numerical result
All medications	Coded (NDC)/RxNorm
All diagnosis – active & inactive	ICD-9
Blood pressure	Systolic and diastolic
Estimated GFR	Calculated value
Urine albumin/creatinine ratio	Calculated value
Medicare insurance coverage	Flag for medicare insurance
Nephrologists referrals	Referral records (when available)

#### ***Process evaluation***

To determine the effectiveness of the TRANSLATE elements, it is crucial to first determine the degree of adoption by the practice, and then systematically document the challenges and enablers of the implementation process, including the role of facilitation and the contextual factors that contribute to success. Qualitative measures will be collected in tandem with the implementation of facilitated TRANSLATE elements to support real-time learning and a reiterative process of evaluation (Table 3).

**Table 3:** Process evaluation activities

**Process evaluation activities**	**When collected**	**Arm(s)**
• Survey of practices' approaches to chronic care and practice change	Baseline Endpoint	Facilitated-CDS and CDS-only
• Semi-structured interviews with each Physician/ Clinician Champion.	Baseline Endpoint	Facilitated-CDS and CDS-only
o Interviews conducted with ALL Facilitated practices		
o Interviews conducted with 9 CDS-only practices (4–5 from each round)		
• Facilitator activity log on all interactions with the practice including:	Ongoing	Facilitated- CDS
o Emails		
o Phone calls		
o Monthly meetings with site coordinator		
o QI team meetings		
o Webinars/Conference calls		
o Collaborative learning		
o Interface/Meetings/consultations with the academic mentors		
• Academic detailing activity log on interactions with the practice including:	Weekly debrief with	Facilitated- CDS
o Phone calls with physicians	each academic mentor	
o Emailsntd	on their contacts with	
o Webinars/Conference calls	the practices	
o Collaborative learning		
o Interface/Meetings/consultations		
• Site visits to 10 interventions and 10 comparator practices to observe workflow.	Final 18 months of	Facilitated-CDS and CDS-only
o Comparator practices selected at random.	intervention	
o Intervention practices selected using a dynamic multi-method approach (refer to		
o site visit protocol)		

Semi-structured interviews will be conducted with the physician champions at baseline and endpoint, asking about their care of patients with CKD and workflow processes in their office. Members of the evaluation team will also conduct site visits at endpoint with both intervention and comparator practices.

E-mail and other communication between practice facilitators and the sites will be analyzed by the evaluation team. Facilitators will also collect notes of meetings, and practice activities; and submit this information to the evaluation team on a regular basis.

#### ***Cost-effectiveness analysis***

The hypothesis is that the intervention of CDS plus the facilitated-TRANSLATE is more cost-effective than the intervention of CDS alone, so the economic analysis will focus on the TRANSLATE elements in delaying CKD progression and reducing mortality due to CKD in the practice settings of primary care physicians. Because there is not a true control group (no clinic practice has no intervention), the proposed project will examine the relative costs of these interventions.

Three types of costs will be included into the total costs of each intervention: the cost of providing services in the clinic practices; the cost of treating CKD; and the other healthcare expenses for patients. We plan to survey a sample of randomly selected practice clinics to measure the cost of providing CKD services. The investigators have created and used a spreadsheet and guideline for collecting cost data from practices for previous studies [39,40]. The practice facilitation activities of TRANSLATE are expected to lead to more costs in the intervention practices. The cost of training clinic practices to use the CDS system and functions of TRANSLATE will also be included into this type of cost, and the cost of training (time) will be measured based on the hourly wages of physician or medical staff. The cost of treating CKD and other healthcare costs for patients will be based on the claims data that we acquire from DARTNet.

### Data analysis

#### ***Clinical data***

The effectiveness measures will include the degree of evidence-based guideline-concordant care for CKD patients (a patient-level score based on the percentage of goals achieved); CKD progression for patients with stage three and four CKD; and all-cause mortality rate.

#### ***Data analysis for hypothesis 1.1 (degree of evidence-based care)***

The primary outcome for this analysis will be a patient-level score based on the percentage of goals achieved. Each goal will be assessed using EHR data for the previous year (or part of the year in which the patient is eligible) at baseline, 12 months, 24 months, and 36 months. Secondary analyses will examine each outcome individually using all available data and continuous measures (*e.g.*, SBP, HbA1c, LDL) or dichotomous measures (ACE/ARB, referral, smoking, NSAIDS). The structure of the data is hierarchical (patients nested within practices) and longitudinal (repeated assessments on patients at baseline, 12, 24, and 36 months).

#### ***Data analysis for hypothesis 2.1 (CKD progression)***

The outcome for this analysis will be eGFR measurements over time. There will be multiple eGFR measures per patient over the duration of the study. We will use general linear mixed-effects models to estimate the rate of decline in eGFR (random intercept, random slope) and the degree to which the baseline covariates predict eGFR. Time for each observation will be coded as days since baseline, converted to months to aid interpretability. The primary hypothesis of difference in slope between treatment groups, adjusting for socio-demographic and clinical covariates, will be tested.

#### ***Data analysis for hypothesis 2.2 (all-cause mortality)***

All-cause mortality will be confirmed using the National Death Index to determine the exact date of death. The outcome for the analysis will be time from baseline to death. Patients who are alive at the end of the study period will be censored at the end of the follow-up time. Assumptions of the proportional hazards model will be checked for each variable. Covariates will include baseline eGFR, defined as the mean of the last two eGFRs prior to study entry, as well as socio-demographic and clinical characteristics.

#### ***Process evaluation***

Qualitative data for the process evaluation will be analyzed using an immersion-crystallization approach [41,42]. This content-driven approach allows the data to speak for itself, as researchers immerse themselves in the data repeatedly to identify themes that emerge [41]. Themes are identified around concepts that are expressed repeatedly in the text and constantly compared back to the data to ensure that they represent the data accurately. Once themes are identified using this method, an additional step in the analysis will be to compare the themes with the elements of the TRANSLATE model to determine how well the data complement the interventional model and how the data can illuminate the process of practice adoption of each element.

#### ***Cost-effectiveness analysis***

Analysis will compare the effectiveness-cost ratios between the two arms to examine which intervention is more cost-effective. Because of the available claims data, electronic medical records data, and mortality data from the National Death Index, the analysis will calculate the quality-adjusted life years (QALYs) lost due to CKD for both interventions. The QALYs will be the most important effectiveness measure in the economic analysis, because QALYs can be valued as a dollar amount. In the United States, $50,000 per QALY is a decision rule that is often used to guide interpretation of cost-effectiveness analyses. We will also do sensitivity analysis by replacing this decision rule with $25,000 or $100,000. We will compare the effectiveness (dollar values of QALYs) with the cost, and determine whether the effectiveness is larger than the cost for each intervention. If the effectiveness is larger than the cost, these interventions will have cost savings. If both interventions will produce cost savings, the proposed project will compare the cost savings for these two interventions and determine which intervention has a high cost saving amount.

### Trial status

Recruitment is ongoing. The first wave of practices was recently randomized, and the official intervention period began these practices in January of 2013. Initial baseline data are being collected (Additional file 1). Data analysis has not yet occurred. We anticipate that we will reach full recruitment (36 practices) and all practices will have entered the intervention (allowing a minimum of 18 months of intervention) by December of 2013. The trial is registered as NCT01767883 on clinicaltrials.gov.

## Discussion

### Limitations

Some data elements like nephrology referral may be difficult to collect if they are entered as free text and not as order entry. The practices will be worked with individually to try and overcome this. We may have difficulty obtaining Medicare claims data in all practices, so we may have to use a sample to complete this. Quality of life measures may be difficult to obtain for cost-effectiveness evaluation. This too, may have to be done with a sub sample. We originally did not block randomize within organizations, but two organizations were found that were highly integrated in how they accomplished QI, so we had to change to block randomization for the groups that had more integrated QI plans, otherwise there would be cross contamination and a dilution of a positive effect of facilitation.

### Innovation and potential impact

This study has three major innovations. First, this study adapts the TRANSLATE method, proven effective in diabetes care [32], to CKD which, by definition, is a complex condition usually presenting with other co-morbidities. Successful application of the TRANSLATE model to complex disease conditions such as CKD will demonstrate the viability of this model for aiding practices in practice transformation and in treatment of patients with other complex co-morbid conditions. Our TRANSLATE framework also incorporates an implementation team that includes a clinician champion, site coordinator, and an administrator to allocate resources and guide and oversee implementation progress. It utilizes information technology systems such as EMR or middleware programs to produce registries to facilitate the identification of high-risk patients and generate performance reports to provide data for ongoing feedback. In future projects, this framework will be used to assess the readiness of practices to participate in QI projects, and to help diagnose why a project may be failing to improve outcomes, thereby allowing for mid-course corrections.

Second, through this study we are creating a generalizable CDS specific to the KDOQI guidelines for CKD: a novel CDS application. The point-of-care CDS protocol engine is integrated with multiple EHRs. The vendor agnostic middleware tools we are using, consisting of CINA and HMS, can be implemented against virtually any ambulatory EHR. The support algorithms have also been adapted and implemented directly into the EHRs. It is hoped that facilitated CDS and registry creation can be an underlying system to rapidly improve evidence-based prevention and chronic disease management with common workflows regardless of condition.

Furthermore, this study will evaluate the effects of CDS versus CDS with facilitation and answer key questions regarding the cost-effectiveness of a facilitated model for improving CKD outcomes. In addition, the study is testing virtual facilitation and Academic detailing making the findings generalizable to any area of the country.

Finally, through the use of DARTNET, the study will further a methodology for tracking in an efficient and longitudinal manner a very large population over a long period of time in real world practices, thereby allowing both group level randomized CRCTs as well as population-based economic analyses to be conducted from the same study.

## Abbreviations

CINA: Computer Integrated Networks of American; CKD: Chronic kidney disease; CDS: Computer decision support; DARTNet: Distributed Area Research and Therapeutics Network; EHR/ EMR: Electronic health record / Electronic medical record; ESRD: End stage renal disease; HMS: Health Metrics Systems; KDOQI: National Kidney Foundation Kidney Disease Outcomes Quality Initiative; PCP: Primary care physician; QALY: Quality adjusted years of life; QI: Quality improvement.

## Competing interests

The authors declare no potential conflicts of interest with respect to the research, authorship, or publication of this article.

## Authors’ contributions

CF is the principal investigator and responsible for the concept and design. BV contributed to the design of the process evaluation and drafted the manuscript. LK designed the process evaluation and reviewed the manuscript critically for content. MD designed the randomization protocol and statistical analysis plan and reviewed the manuscript critically for content. HF designed the cost-effectiveness analysis and reviewed the manuscript critically for content. WP contributed to the content and design of the study and reviewed the manuscript critically for content. KK contributed to the design of the facilitation intervention and the process evaluation and reviewed the manuscript critically for content. JV contributed to the content and design of the study and reviewed the manuscript critically for content. NL contributed to the overall protocol development and standardization and reviewed the manuscript critically for content. KP contributed to the concept and design of the study. All authors read and approved the final manuscript.

## Supplementary Material

Additional file 1: Table S1 Consort 2010 checklist of information to include when reporting a cluster randomised trial. Click here for file
